# Desmoid-type fibromatosis mimicking cystic retroperitoneal mass: case report and literature review

**DOI:** 10.1186/s12880-018-0265-5

**Published:** 2018-09-17

**Authors:** Kyu-Chong Lee, Jongmee Lee, Baek Hui Kim, Kyeong Ah Kim, Cheol Min Park

**Affiliations:** 1 Department of Radiology, Korea University Guro Hospital, Korea University College of Medicine, 80 Guro-dong, Guro-gu, Seoul, 152-703 South Korea; 2 Department of Pathology, Korea University Guro Hospital, Korea University College of Medicine, Seoul, South Korea

**Keywords:** Desmoid-type fibromatosis, Desmoid tumor, Retroperitoneum, Ultrasonography, Computed tomography

## Abstract

**Background:**

Retroperitoneal desmoid-type fibromatosis (DF) is an uncommon mesenchymal neoplasm presenting as a firm mass with locally aggressive features. It usually manifests as a well-circumscribed or ill-defined, solid mass on cross-sectional imaging. Cystic changes of DF have been described in the literature in association with prolonged medical treatment or abscess formation. However, spontaneous cystic change is rarely reported.

**Case presentation:**

Here we report the case of a 46-year-old patient with a DF mimicked a large cystic tumor in the retroperitoneum. Ultrasonography and computed tomography were performed in order to search for localizations and characteristics of the cystic tumor. Radiological findings showed an oval cystic mass with a relatively thick wall, measuring 18.3 × 12.3 × 21.5 cm in the left upper abdomen. Laparoscopic spleen-preserving distal pancreatectomy was performed and histopathological examination by immunohistochemical study enabled us to diagnose a DF invading the pancreatic parenchyma. The patient remained asymptomatic during an 8-month follow up period.

**Conclusions:**

We report an extremely rare case of retroperitoneal DF with spontaneous cystic change. DF can manifest as a mainly cystic mass with a thick wall, as in our case, which makes the correct diagnosis difficult. DF should be included in the preoperative differential diagnosis of a cystic retroperitoneal mass, regardless of its rarity.

## Background

Desmoid-type fibromatosis (DF), also called desmoid tumor or deep fibromatosis, is an uncommon mesenchymal neoplasm composed of fibrous soft-tissue proliferation. The tumor is characterized by locally aggressive growth and frequent recurrence, although it never metastasizes [1]. DF usually occurs sporadically, but approximately 5% arise in association with familial adenomatous polyposis (FAP) [2]. Retroperitoneal DF is rare and accounts for less than 1% of retroperitoneal masses [3].

DF usually presents as a well-circumscribed solid mass on imaging studies [4]. Cystic changes of DF are rare and a few reports have suggested an association with prolonged medical treatment or abscess formation [5, 6]. Spontaneous cystic degeneration in DF is extremely rare and only a few reports of such masses have been published [7–9]. Most of them were small lesions (less than 10 cm) presenting as pancreatic or mesenteric cystic tumors.

We report here an unusual case of sporadic retroperitoneal DF with spontaneous cystic change mimicking a cystic tumor, including histopathologic correlations.

## Case presentation

**Fig. 1:**
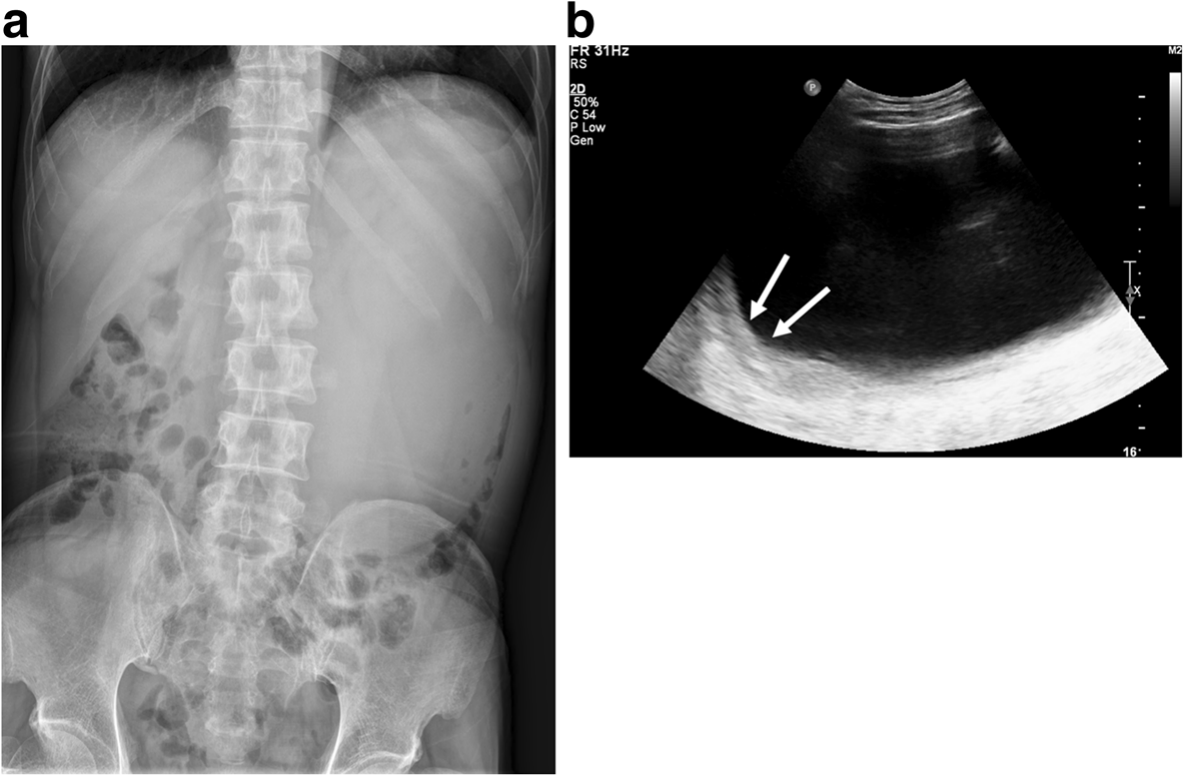
Retroperitoneal desmoid-type fibromatosis in a 46-year-old man. **a** Abdominal plain radiography shows a large mass-like opacity in the left abdomen. **b** The ultrasonography shows a large anechoic cystic mass with a thick wall (arrows) without an intracystic solid portion or septum in the left abdomen

**Fig. 2:**
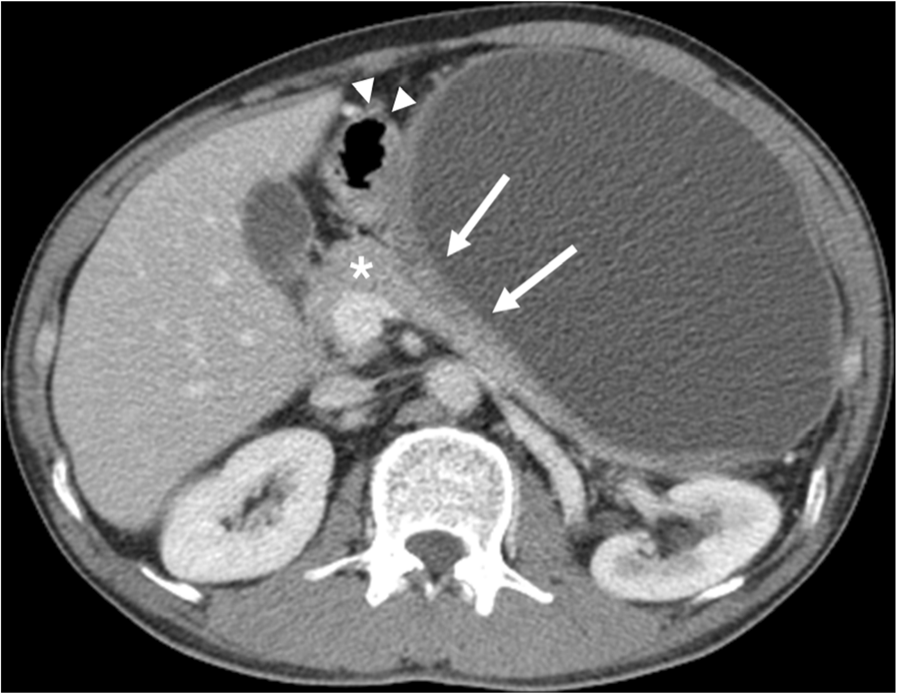
Retroperitoneal desmoid-type fibromatosis in a 46-year-old man. Axial image of CT shows an 18.3 × 12.3 × 21.5 cm sized oval cystic mass with a thick wall (arrows) that has a mass effect on the stomach (arrowheads) and pancreas (asterisk). There is no evidence of an enhancing septum or a solid portion within the cystic mass

A 46-year-old man visited the emergency department of our institution due to left lower quadrant pain and a palpable mass in the left upper abdomen. He had no specific relevant past medical history or family history. Physical examination disclosed a large, tender mass in the left abdomen. All laboratory findings were within normal ranges except a slightly increased CRP level (5.82 mg/L). Abdominal plain radiographs showed a large mass-like opacity in the left abdomen (Fig. 1a). Ultrasonography revealed a large, thick-walled cystic mass without evidence of an intracystic solid portion or septum (Fig. 1b). The patient underwent computed tomography (CT) scans to evaluate the intra-abdominal mass using a 64-slice multidetector CT scanner. Contrast-enhanced CT images revealed an 18.3 × 12.3 × 21.5 cm sized oval cystic mass with a relatively thick wall in the left upper abdomen (Fig. 2). This lesion caused an extrinsic mass effect on the adjacent stomach and pancreas. The boundary between the mass and adjacent pancreas parenchyma was indistinct. Based on these imaging findings, a neurogenic tumor with cystic changes, a mucinous cystadenoma, and a pseudocyst were considered in the differential diagnoses. The patient underwent laparoscopic spleen-preserving distal pancreatectomy without preoperative biopsy due to a risk of rupture.

**Fig. 3:**
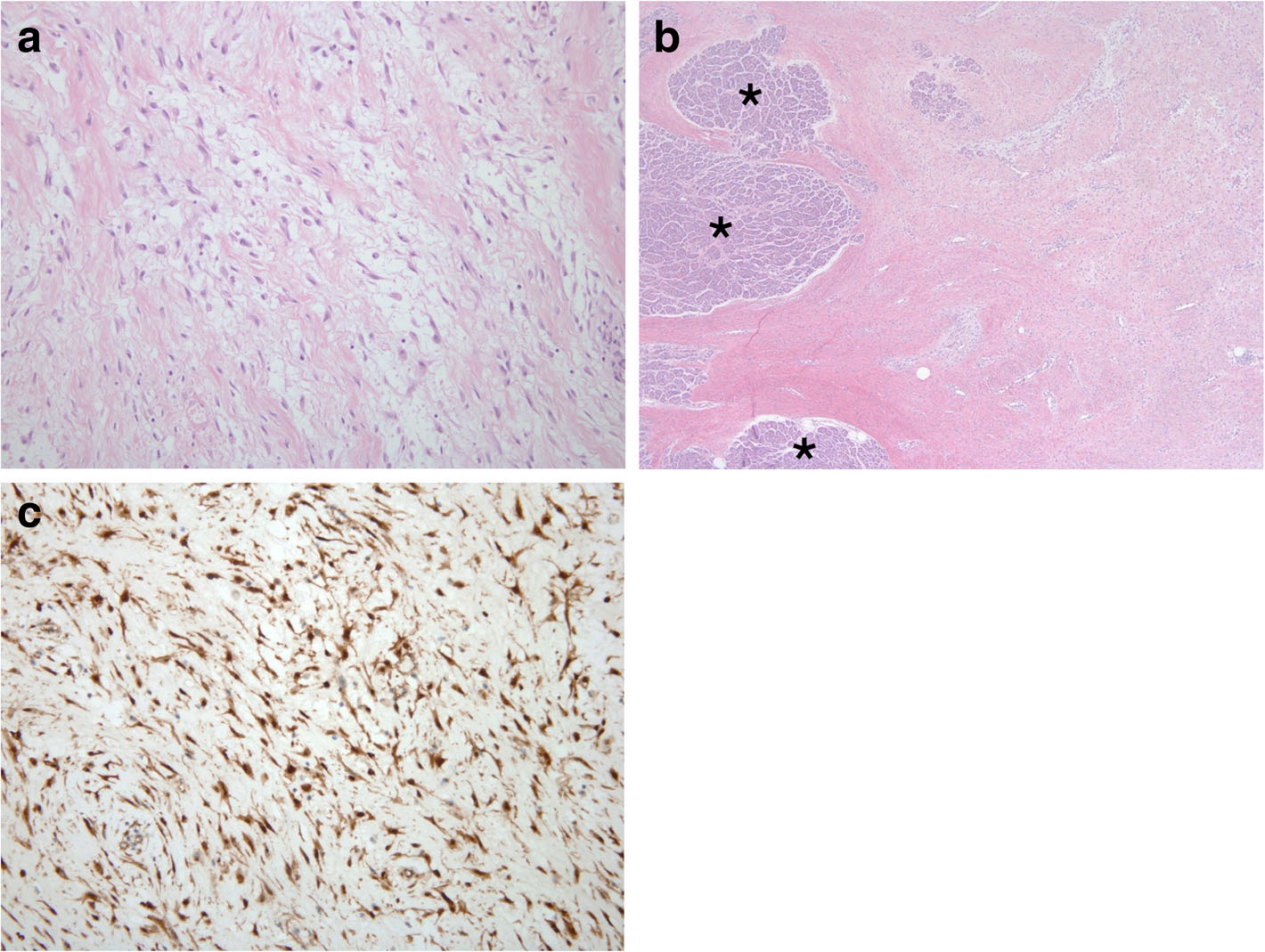
Retroperitoneal desmoid-type fibromatosis in a 46-year-old man. **a** Microscopically, the tumor is composed of uniform, elongated spindle-shaped cells within a collagenous stroma. Spindle cells show mild nuclear pleomorphism and no mitotic figures (hematoxylin and eosin stain; original magnification × 100). **b** On a lower power view, tumor cells are diffusely infiltrating into the pancreatic parenchyma (asterisks) (hematoxylin and eosin stain; original magnification × 40). **c** Immunohistochemical study of the tumor shows strong nuclear and cytoplasmic positivity for intranuclear β-catenin (β-catenin; original magnification × 100)

Intra-operatively, the mass was confirmed to have arisen from the retroperitoneum, closely related to the pancreas tail. Surgeons found hemorrhagic fluid within the cystic mass. The surgical specimen was a large round lump of soft tissue measuring 13 × 10.5 × 4.3 cm in size. On gross section, the cut surface revealed a rubbery texture with a whitish to light yellowish color. Almost half of the mass was composed of a cystic space that was filled with clear, light brownish fluid. The mass was diffusely infiltrating the pancreatic parenchyma. Microscopically, the tumor was composed of uniform sheets of elongated, spindle-shaped cells in a collagenous stroma (Fig. 3a). The tumor was intermingled with the pancreatic parenchyma (Fig. 3b). Immunohistochemical study showed the tumor cells were positive for smooth muscle actin (SMA) and beta-catenin (Fig. 3c), but negative for S-100 protein and CD34. The final pathologic diagnosis was DF. The postoperative course was uneventful, and the patient was discharged on postoperative day 20. The patient remained asymptomatic during an 8-month follow up period.

## Discussion

The term “fibromatosis” describes a group of conditions that consist of fibroblastic proliferation in a collagenous extracellular matrix. Fibromatosis can be either superficial (fascial) or deep (musculoaponeurotic). In 2002, the World Health Organization (WHO) used the term ‘desmoid-type fibromatosis’ for deep fibromatosis [10], which is usually called a desmoid tumor. It can be further classified on the basis of its anatomic location, such as abdominal wall, intra-abdominal, or extra-abdominal. In various studies, 28 to 69% of DF was intra-abdominal (mesenteric or pelvic) or located in the abdominal wall [11]. Retroperitoneal DF accounts for less than 1% of retroperitoneal masses [3]. Multiple risk factors for DF are widely known, including genetic mutations of the adenomatous polyposis coli (APC) gene such as in FAP or the beta-catenin gene (CTNNB1), previous surgery, trauma, pregnancy, and oral contraceptive use [11, 12]. However, the pathogenesis of DF is not completely understood.

DF is more common in young women, from puberty to 40 years of age [1]. However, intra-abdominal fibromatosis shows no gender difference or age predilection [10]. Most patients present with an asymptomatic abdominal mass, but some have mild abdominal pain. Although less common, patients with mesenteric lesions are accompanied by gastrointestinal bleeding or acute abdominal pain secondary to bowel perforation [4].

Histologically, DF is composed of elongated, uniform spindle cells within a collagenous stroma. Although DF appears well-delineated at gross analysis and cross-sectional imaging, at the microscopic level its margins appear to infiltrate the adjacent structures. Immunohistochemically, the tumor cells are negative for CD34, CD117, and S-100 protein [1]. These findings exclude gastrointestinal stromal tumors and neurogenic tumors. Immunoreactivity for β-catenin supports the diagnosis of DF but is not pathognomonic for this disease because other entities, including superficial fibromatosis, low-grade myofibroblastic sarcomas, and solitary fibrous tumors, may also exhibit nuclear staining for β-catenin [13].

DF usually appears as a well-defined solid mass on imaging studies. The ultrasonographic appearance of DF is a solid, usually well-circumscribed and hypoechoic mass of variable vascularity [4, 11]. On CT, DF appears as a soft tissue mass of variable attenuation and enhancement, which depends on tissue components [3, 4]. DF with a highly collagenous stroma usually displays homogeneous, soft-tissue attenuation on CT scans. DF with a myxoid matrix appears as a hypoattenuating lesion. Some lesions may appear striated or whorled because of the alternating collagenous and myxoid area. Heterogeneous attenuation may be seen due to necrosis or degeneration. The soft-tissue component is such a dominant feature that it often appears similar to solid tumors, such as gastrointestinal stromal tumor, lymphoma, or soft tissue sarcoma [4]. DF has invasive properties and tends to damage blood vessels.

Cystic changes of DF have been rarely reported and an association with prolonged medical treatment or abscess formation has been suggested [5, 6]. However, these causal relationships are irrelevant in this case because our patient was male and there was no evidence of abscess formation in DF on histopathologic examination. Tan et al. [9] postulated that cystic appearance of DF was the result of spontaneous tumor regression. Spontaneous regression of DF is thought to be related to the withdrawal of estrogenic stimulation and could also result from secondary infarction of either superimposed infection or compromised vascular supply to the tumor. DF has an invasive nature and tends to cause vascular compromise. Furthermore, ischemia during rapid tumor growth may be the cause of extensive cystic degeneration of DF. An infarction secondary to a compromised vascular supply may be a possible cause of cystic degeneration in DF in our case. However, it is difficult to explain this extensive cystic change.

In the present case, cystic DF invasion to the pancreas was confirmed by pathology. Only 13 cases have been reported of intra-abdominal DF involving the pancreas [8, 14]. Nine out of 13 cases involved the pancreatic tail, similar to our case. For DF involving the pancreas, which usually appears as a solid mass, only two cases have been considered purely cystic and four cases appeared as mixed cystic and solid masses. DF begins as small scar-like foci of fibrosis in the retroperitoneal fat and, when large, it typically spreads around and between other structures [15]. Therefore, retroperitoneal DF may involve the pancreas and can be interpreted as a pancreas-originating lesion.

A multidisciplinary approach can help patients with DF receive optimal management. Stable, asymptomatic DF can be observed. However, treatment is necessary for symptomatic subjects like our patient. If feasible, surgical excision is regarded as the conventional treatment of choice. However, recurrence is common. The recurrence rate is 15–30% for intra-abdominal DF [11].

## Conclusion

We report an extremely rare case of retroperitoneal DF with spontaneous cystic change. It can manifest as a mainly cystic mass with a thick wall, as in our case, that makes it difficult to reach a correct diagnosis. DF should be included in the preoperative differential diagnosis of a cystic retroperitoneal mass, regardless of its rarity.

## Abbreviations

APC: Adenomatous polyposis coli
CRP: C-reactive protein
CT: Computed tomography
DF: Desmoid-type fibromatosis
FAP: Familial adenomatous polyposis
SMA: Smooth muscle actin
